# METABOLIC SYNDROME PREVALENCE AND CARDIOVASCULAR DISEASES RISK AMONG SCHOOL TEACHERS

**DOI:** 10.13075/ijomeh.1896.02584

**Published:** 2025

**Authors:** Mona Abdallah Ramadan, Aisha Safwat Saif Eldeen, Basma Hussein Mourad

**Affiliations:** 1 Cairo University, Faculty of Medicine, Department of Occupational and Environmental Medicine, Cairo, Egypt; 2 Princess Nourah bint Abdulrahman University, Department of Health Sciences, College of Health and Rehabilitation Sciences, Riyadh, Saudi Arabia

**Keywords:** hypertension, diabetes, dyslipidemia, metabolic syndrome, school teachers, CVD risk

## Abstract

**Objectives:**

Educators face a wide range of recognized biological, physical, and other workplace hazards making them more susceptible to increasing the prevalence of metabolic syndrome (MetS) and associated cardiovascular diseases (CVDs) risks. The current research aimed to evaluate the prevalence of MetS and the likelihood of CVDs among school teachers.

**Material and Methods:**

A cross-sectional study was conducted on 281 participants chosen from schools in the El-Maadi region of Cairo, Egypt. Socio-demographic, occupational, and medical data were collected. Standard procedures were employed to assess fasting blood glucose (FBG), and lipid profile. Metabolic syndrome was defined using criteria from the International Diabetes Federation (IDF). The 10-year atherosclerotic cardiovascular disease (ASCVD) risk was estimated using the ASCVD risk score estimator as per the 2019 American College of Cardiology and American Heart Association guideline.

**Results:**

Metabolic syndrome had an overall prevalence rate of 25.8%. Among the instructors evaluated, 73% had a low 10-year risk of getting CVDs, whereas 3.1% had a high risk. The study identified significant correlations between the prevalence of MetS and many characteristics, including age, marital status, length of job, level of education, smoking, prevalence of diabetes and hypertension, central obesity, measured blood pressure, FBG levels, and dyslipidemia among the participants.

**Conclusions:**

School teachers exhibit a considerable prevalence of MetS and risk of CVDs. Health promotion activities and stress management interventions should be implemented.

## Highlights

- Metabolic syndrome prevalence among studied teachers was 25.8%.
- Ten-year cardiovascular diseases risk: low 73%, borderline 8.8%, intermediate 15.1% and high 3.1%.
- Key risk factors: age, marriage, work duration, education, smoking, diabetes mellitus and obesity.
- Promotion of healthy behaviors, such as a balanced diet and regular exercise, is essential for teachers.
- School health programs should address teacher stress and overall health needs.

## INTRODUCTION

Noncommunicable diseases (NCDs) are widely acknowledged as the primary cause of sickness on a global scale. Cardiovascular diseases (CVDs) were responsible for the bulk of the 42 million deaths globally in 2018, totaling 18.6 million. According to the WHO, the number of individuals affected with diabetes is estimated to reach 425 million [1,2]. In Egypt, the annual death rate from CVDs is 40%, and 20.9% of people aged 20–79 years have diabetes, which caused 122 684 fatalities in 2021 [3,4].

Metabolic syndrome (MetS) is becoming more common worldwide and is connected to a greater risk of many NCDs, including CVDs and type 2 diabetes mellitus (T2DM) [5]. These conditions are associated with increased rates of illness and death, as well as large healthcare expenses [6]. Prior research has shown that patients with MetS have a 2 times higher risk of developing cardiovascular illnesses and a 5 times higher chance of developing T2DM [7,8].

Metabolic syndrome is a collection of risk factors for CVDs and T2DM. These risk factors include high blood sugar levels (hyperglycemia), abnormal lipid levels (dyslipidemia), high blood pressure (BP) (hypertension), and obesity. The condition is correlated with a substantial susceptibility to CVDs and is projected to impact a min. 20% of the worldwide population [9]. The adult population of Egypt has a very high incidence of MetS, standing around 55% [10].

The diagnostic criteria for MetS include elevated BP, plasma triglycerides, obesity (as measured by waist circumference [WC] or body mass index [BMI]), high-density lipoprotein (HDL) cholesterol, and fasting blood sugar (FBS) levels [11]. According to the National Cholesterol Education Program Adult Treatment Panel III (NCEP ATP III), an individual is diagnosed with MetS if ≥3 of these 5 components exceed predefined clinical thresholds [12]. A combination of genetic, metabolic, behavioral (especially related to diet and exercise), cultural, environmental, and socioeconomic factors contributes to the development of MetS. The increase in lifestyle-related illnesses, including CVDs and MetS, has been influenced by changes in the working environments of a variety of professional groups, particularly school instructors [8,13].

In numerous countries, educators comprise a substantial and expanding proportion of the labor force. Teaching is one of the most stressful professions, characterized by high levels of stress, burnout, and absenteeism. Educators face a wide range of recognized biological, physical, and other workplace hazards [14].

Teacher stress can stem from various sources depending on the grade level taught. These sources include issues related to curriculum and administration, job progression, extensive verbal communication, work overload due to large class sizes, student motivation, and prolonged standing. Teachers often experience role conflict, job security concerns, and overwhelming responsibilities, especially under extreme time constraints. In addition to physical or environmental risks, such as noise, stress can arise from managing student misbehavior [15].

While numerous studies have investigated the prevalence of MetS and CVD risk in the general adult population worldwide, no published data is available on Egyptian school teachers. Therefore, the current study investigated the prevalence of MetS and the risk of CVDs among Egyptian school teachers, facilitating early identification, appropriate management, and effective control of related illnesses.

## MATERIAL AND METHODS

A cross-sectional study was conducted on school teachers in the El-Maadi region of Cairo, Egypt, in January–May 2024. The available population was 500 teachers after applying the inclusion and exclusion criteria. Those aged ≥40 years, with at least 5 years of continuous working experience in this occupation without leave, were included in the study. Teachers were excluded if they had CVD, were pregnant, taking low-density lipoprotein (LDL)-lowering medication, refused to participate in any research procedures or were ill at the time of the study. Based on estimates of the sample size, 281 teachers out of the 500 were selected using the Microsoft Excel (simple random technique) to participate in the study.

### Sample size

In order to evaluate the prevalence of MetS among Egyptian school instructors, the sample size was calculated to find the minimum appropriate number. A literature review indicated that prevalence in similar contexts ranged 22–29% [9,16], with an average of mean (M) ± standard deviation (SD) 25.5±3.5%. Assuming this is the true population prevalence, a study of 255 participants is required to achieve 95% confidence interval (CI), with a 6% margin of error, using the generic Z test. The sample size was increased by 10% to 281 participants (instead of 255) to account for potential dropouts. To calculate the sample size StatCalc, Epi Info v. 7.2.5 for MS Windows (Centers for Disease Control and Prevention, Atlanta, USA) was employed.

### Methodology

The research strategy and testing protocols were communicated to each participant, and after receiving full information, each individual gave informed written permission. The Ethical Committee of the Faculty of Medicine, Cairo University, Egypt, approved the study (N-79-2024). The study was executed in adherence to the ethical principles outlined in the Declaration of Helsinki in October 2013.

All participants were subjected to the following procedures: interview questionnaire, anthropometric measurements and BP measurement.

#### Interview questionnaire

The interview questionnaire included socio-demographic history (age, marital status, smoking habits, medical history, and anthropometric measurements) and occupational history (working hours per day, working days per week, years of employment, teaching stage, additional jobs, and previous employment).

#### Anthropometric measurements

The weight was measured using a handheld digital scale and reported to the closest 0.1 kg.

Height was obtained with precision to the closest 0.1 cm using a measuring tape affixed to a wall.

The BMI was determined by dividing the weight of the individual in kilograms by the square of the height in meters and indicated as:

– underweight for BMI <18.5 kg/m^2^,
– normal weight range for BMI of 18.5–24.9 kg/m^2^,
– overweight for BMI of 25–29.9 kg/m^2^,
– obesity for BMI ≥ 30 kg/m^2^.

These classifications are based on the 2005 guidelines of the WHO [17].

Waist circumference was measured using a rigid plastic measuring tape that does not stretch. It was positioned half an inch above the umbilicus, and the measured values were rounded to the closest 0.1 cm. Abdominal obesity is characterized by a ≥94 cm WC for males and ≥80 cm for women [18].

#### Blood pressure

The participants' BP was assessed using a sphygmomanometer after sitting and resting for a min. 5 min, with their arms placed on a table. Two measurements were obtained during a single visit, and the average was utilized as the final value. Participants were directed to refrain from smoking, consuming coffee or tea, and participating in any physical activity for a min. 30 min before the assessment. Hypertension was diagnosed if the diastolic BP was ≥90 mm Hg and/or the systolic BP was ≥140 mm Hg, in accordance with national standards [19].

### MetS definition

The definition of MetS was established based on the criteria provided by the International Diabetes Federation (IDF). Participants were classified as being at risk for MetS if they exhibited triglyceride levels ≥150 mg/dl, HDL cholesterol levels <40 mg/dl for men or <50 mg/dl for females, fasting blood glucose (FBG) levels ≥110 mg/dl, or BP ≥130/85 mm Hg. Central obesity is characterized by a WC of ≥94 cm in males and ≥80 cm in women. Participants were identified with MetS if they fulfilled ≥3 of the preceding requirements [20].

### CVD risk assessment utilizing the Atherosclerotic Cardiovascular Disease (ASCVD) Risk Estimator

The ASCVD Risk Estimator is designed to be used in conjunction with the 2018 American College of Cardiology and the American Heart Association (ACC/AHA) Guideline on the Management of Blood Cholesterol and the 2013 ACC/AHA Guideline on the Assessment of Cardiovascular Disease Risk [21]. By using the Pooled Cohort Equations and lifetime risk prediction tools, this Risk Estimator helps patients and healthcare professionals to estimate their 10-year and lifetime risks for ASCVD, which is defined as coronary death or nonfatal myocardial infarction or fatal or nonfatal stroke. Arnett et al. established a guideline that offers a scientifically supported strategy for managing risk factors comprehensively in order to decrease the occurrence of CVD [21]. To calculate the 10-year risk score for ASCVD, you need to provide information on gender, age, race, BP, cholesterol profile, antihypertensive therapy, history of T2DM, and smoking history. The findings were classified into 4 risk categories:

– minimal risk (<5%),
– the danger level is considered borderline when it falls between 5% and 7.4%,
– the risk level is considered intermediate when it falls between 7.5% and 19.9%,
– elevated risk (≥20%).

### Sample collection and analysis

Each participant was instructed to fast for 12 h and refrain from exercise or smoking before blood sampling. A 5 ml peripheral blood sample was drawn from each participant via venipuncture using a sterile, disposable syringe in the early morning. The sample was placed in a plain tube for centrifugation and serum separation to measure FBS, total serum cholesterol, triglyceride levels, and HDL cholesterol using enzymatic colorimetric tests. Total cholesterol, HDL cholesterol, and triglyceride levels were used to estimate serum LDL cholesterol [22].

### Statistical methods

Data were coded and inserted into the SPSS software v. 28 (IBM Corp., Armonk, USA). The test known as the Shapiro-Wilk was implemented to evaluate the degree of normality of the data. The authors used either M±SD or median (range) to report numerical variables. To compare the 2 groups, the Mann-Whitney test for non-normally distributed variables and the unpaired t-test for normally distributed variables were employed [23]. Frequencies (numbers) and relative frequencies (percentages) were reported for categorical variables. The exact test was employed when the anticipated frequency was <5, and the chi-square (χ^2^) test was employed to compare categorical data [24]. A correlation between quantitative variables was determined using Spearman's correlation coefficient [25]. Linear regression analysis was conducted to identify the statistically significant independent predictors of the ASCVD score using all the socio-demographic, occupational, and medical variables of the studied teachers[26]. Statistical significance was determined by p < 0.05.

## RESULTS

The study sample comprised 281 teachers aged 40–59 years, with age of M±SD 47.26 ± 5.75 years. Of the participants, 30.2% were >50 years, while 69.8% were 40–50 years old. The sample included 56.6% females and 43.4% males, with 87.5% being married. Regarding their teaching background, 29.6% taught at the primary level, 33.3% at the preparatory level, and 37.1% at the secondary level. Teaching experience ranged 14–39 years, with 8–14 working hours/day and 5–7 working days/week.

Regarding educational attainment, 85.5% of the teachers held a university degree, and 14.5% had a postgraduate degree. Less than one-fourth (24.5%) of the teachers were smokers, with a median (Me) = 22 pack/year.

The study found that 59.1% of the teachers exhibited a WC exceeding the typical range, with M±SD 100.35±15.32 cm, and 88.78±16.34 cm across males and females, respectively. The normal WC is <94 cm in males and <80 cm in females. Regarding BMI, 34.6% of the respondents were classified as obese (BMI >30), with BMI M±SD 28.95±4.48, and 28.89±5.23 kg/m^2^ for males and females, respectively. Only 11.3% and 11.9% of the teachers had a history of diabetes and hypertension, respectively. Blood pressure measurements during the study indicated that 37.7% of the participants had elevated BP (≥140/90 mm Hg), while 62.3% had normal BP. The M±SD of BP measurements, FBS levels, and lipid profile figures for the group are also shown (Table 1 and 2).

**Table 1.** Mean, standard deviations, and ranges of some personal, occupational, and medical data among the studied teachers (N = 281), Cairo, Egypt, January–May 2024

Variable	M	SD	Min.	Max	Me	Range
Socio-demographic and behavioral						
age [years]	47.26	5.75	40.00	59.00		
duration of employment [years]	25.35	6.16	14.00	39.00		
working time						
h/day	11.65	1.53	8.00	14.00		
days/week	5.90	0.36	5.00	7.00		
smoking [pack/year]					22	0–40
Medical						
BMI [kg/m^2^]						
male	28.95	4.48	21.50	46.70		
female	28.89	5.23	20.20	46.70		
waist circumference [cm]						
male	100.35	15.32	71.0	140.00		
female	88.78	16.34	62.00	129.00		
blood pressure [mm Hg]						
systolic	131.73	21.01	90	170		
diastolic	83.63	11.51	60	110		
fasting blood sugar [mg/dl]	101.14	21.96	62.00	166.00		
cholesterol [mg/dl]						
total	195.33	41.13	125.00	316.00		
HDL	51.13	12.49	20.50	94.70		
LDL	116.30	45.63	36.00	248.30		
triglycerides	145.97	29.34	76.10	199.00		
atherosclerotic cardiovascular disease risk score					2.2	0.1–38.8

**Table 2.** Frequencies of some socio-demographic, occupational, and medical data in addition to the prevalence of metabolic syndrome and atherosclerotic cardiovascular disease (ASCVD) risk categories among the studied teachers, Cairo, Egypt, January–May 2024

Variable	Participants (N = 281)	
	n	%
Socio-demographic and behavioral		
age group		
40–50 years	196	69.8
>50 years	85	30.2
sex		
male	122	43.4
female	159	56.6
marital state		
married	246	87.5
single	16	5.7
widow	9	3.2
divorced	10	3.6
smoking (yes)	69	24.5
Occupational		
educational level		
university	240	85.5
postgraduate	41	14.5
stage of teaching		
primary	83	29.6
preparatory	94	33.3
secondary	104	37.1
Medical		
history of diabetes (yes)	32	11.3
history of hypertension (yes)	33	11.9
BMI >30 kg/m^2^ (yes)	97	34.6
increased waist circumference (yes)	166	59.1
increased blood pressure measurements (yes)	106	37.7
metabolic syndrome (yes)	72	25.8
ASCVD risk		
low	205	73.0
borderline	25	8.8
intermediate	42	15.1
high	9	3.1

According to the criteria of the IDF, 25.8% of the teachers were diagnosed with MetS. The 10-year ASCVD risk score, calculated according to the 2018 ACC/AHA guidelines, showed that 73% of the teachers were at low risk, 8.8% at borderline risk, 15.1% at intermediate risk, and 3.1% at high risk. The ASCVD risk score was Me = 2.2 (Table 1).

A statistically significant higher prevalence of MetS was observed among teachers >50 years old (54.1%) compared to those aged 40–50 years (13.3%). Female teachers had a slightly lower prevalence of MetS (25.2%) compared to males (26.2%), and this difference was not statistically significant. Married teachers had a statistically significant higher prevalence of MetS (26.4%) compared to unmarried teachers (20%).

Teachers with >20 years of experience had a significantly higher prevalence of MetS (31.1%) compared to those with ≤20 years (p = 0.021). Teachers with a postgraduate degree had a significantly higher prevalence of MetS (53.7%) compared to those with only a university degree (20.8%). While no statistically significant difference in MetS prevalence was found across different teaching stages, teachers at the secondary level had the highest prevalence (31.7%). Diabetic and hypertensive teachers had significantly higher MetS prevalence rates (84.4% and 78.8%, respectively) compared to non-diabetic and non-hypertensive individuals. Similarly, teachers with a BMI >30 kg/m^2^ and/or increased WC had significantly higher MetS prevalence rates (61.9% and 39.8%, respectively) compared to those with a BMI <30 kg/m^2^ and/or normal WC. Teachers with elevated BP (either diagnosed hypertensive or not) had a significantly higher MetS prevalence (51.9%) compared to those with normal BP measurements.

Significant associations were observed between the occurrence of MetS and the following factors: age, years of teaching, number of working days/week, number of working hours/day, number of smoked packs of cigarettes/year, BMI, BP, FBS, and lipid profile (Table 3).

**Table 3.** The association between the diagnosis of metabolic syndrome (MetS) among the studied teachers (N = 281) and their socio-demographic, occupational, and medical data, Cairo, Egypt, January–May 2024

	Participants [n (%)]		p	M±SD		p
	with MetS	without MetS		with Me	without MetS	
Socio-demographic and behavioral						
age [years]			0.013^*^	50.12±5	46.26±5.34	**<0.001**
40–50 years	26 (13.3)	170 (86.7)				
>50 years	46 (54.1)	39 (45.9)				
sex			0.939			
male	32 (26.2)	90 (73.8)				
female	40 (25.2)	119 (74.8)				
marital status			**0.047**			
married	65 (26.4)	181 (73.6)				
unmarried	7 (20)	28 (80)				
smoking [packs/year]				9.8±23.	9 7.97±15.88	**0.048**
Occupational						
duration of employment [years]			**0.021**	28.59±6	2 24.23±5.75	**<0.001**
≤20 years	12 (13.6)	76 (86.4)				
>20 years	60 (31.1)	133 (68.9)				
educational level			**0.015**			
university	50 (20.8)	19079.2)				
postgraduate	22 (53.7)	19 (46.3)				
stage of teaching			0.063			
primary	16 (19.3)	67 (80.7)				
preparatory	23 (24.5)	71 (75.5)				
secondary	33 (31.7)	71 (68.3)				
working time						
h/day				12.24±1	11.44±1.567	**0.004**
days/week				6±0.31		**0.037**
Medical						
history of diabetes (yes)	27 (84.4)	5 (15.6)	**<0.001**			
history of hypertension (yes)	26 (78.8)	7 (21.2)	**<0.001**			
BMI						
kg/m^2^				36.6±3.	1 25.8±4.55	**0.018**
>30 kg/m^2^ (yes)	60 (61.9)	37 (38.1)	**<0.001**			
increased waist circumference (yes)	66 (39.8)	100 (60.2)	**<0.001**			
increased blood pressure measurements (yes)	55 (51.9)	51 (48.1)	**<0.001**			
blood pressure [mm Hg]						
systolic				144.85±2	.68 127.3±16.74	**<0.001**
diastolic				89.68±10	09 81.61±11.32	**<0.001**
fasting blood sugar [mg/dl]				108.20±22.63	98.69±21.28	**0.017**
cholesterol [mg/dl]						
total				213.78±40.81	188.92±39.42	**0.001**
HDL				44.81±12.37	53.33±11.81	**<0.001**
LDL				138.89±41.22	108.45±44.61	**<0.001**
triglycerides				164.87±26.15	139.40±27.57	**<0.001**

HDL – high-density lipoprotein; LDL – low-density lipoprotein.

Bolded are statistically significant p-value.

Atherosclerotic cardiovascular disease score components only include systolic and diastolic BP, cholesterol, and HDL; however, in this manuscript, the authors include all studied variables to test the relationship between studied variables and ASCVD risk.

Spearman correlation analysis showed positive correlations between the ASCVD risk score and age, years of employment, number of working days/week, number of working hours/day, number of smoked packs of cigarettes/year, BMI, systolic BP, diastolic BP, FBS, cholesterol, triglycerides, and LDL levels. A negative correlation was observed between the ASCVD risk score and HDL levels (Table 4).

**Table 4.** Spearman's correlations between the atherosclerotic cardiovascular disease (ASCVD) risk score among the studied teachers (N = 281) and their socio-demographic, occupational, and medical data (N = 281), Cairo, Egypt, January–May 2024

Variable	Spearman's correlation	
	r	p
Socio-demographic and behavioral		
age	0.6	**<0.001**
duration of employment	0.614	**<0.001**
working time		
h/day	0.353	**<0.001**
days/week	0.374	**<0.001**
smoking	0.557	**<0.001**
Medical		
BMI	0.481	**0.013**
waist circumference	0.473	**0.031**
blood pressure		
systolic	0.373	**<0.001**
diastolic	0.311	**<0.001**
fasting blood sugar	0.184	**0.020**
cholesterol		
total	0.490	**<0.001**
HDL	–0.465	**<0.001**
LDL	0.545	**<0.001**
triglycerides	0.337	**<0.001**

HDL – high-density lipoprotein; LDL – low-density lipoprotein.

Bolded are statistically significant p-value.

Using all the obtained socio-demographic, occupational, and medical data of the studied teachers, linear regression analysis identified smoking (pack/year), duration of employment in years, serum LDL, and FBS as the statistically significant independent predictors of the ASCVD risk (Table 5).

**Table 5.** The statistically significant independent predictors of atherosclerotic cardiovascular disease (ASCVD) risk revealed by linear regression analysis of the socio-demographic, occupational, and medical data of the studied teachers (N = 281), Cairo, Egypt, January–May 2024

Predictor	B	SE	β	t	p	95% CI for B
Constant	−12.761	1.913		−6.672	**<0.001**	−16.539–(−8.982)
Smoking	0.176	0.018	0.512	9.645	**<0.001**	0.140–0.211
Duration of employment	0.301	0.055	0.300	5.512	**<0.001**	0.193–0.409
Low-density lipoprotein (LDL)	0.040	0.007	0.294	5.549	**<0.001**	0.026–0.054
Fasting blood sugar	0.034	0.015	0.121	2.285	**0.024**	0.005–0.064

β – standarized coefficient; B – unstandarized coefficient; SE – standard error.

Bolded are statistically significant p-value.

## DISCUSSION

The present study involved 281 Egyptian teachers from schools in the El-Maadi region of Cairo, Egypt, where participants face workplace stress, the consequences of urbanization, and dietary changes, such as heightened availability of processed meals, inconsistent meal timings, and decreased levels of physical exercise. The objective of this investigation was to determine the prevalence of MetS and the corresponding risk of CVDs among the Egyptian school instructors who were surveyed. The study results revealed an overall MetS prevalence of 25.8% (72 subjects) among the participants. Similarly, a recent cross-sectional study among 216 urban South African school teachers reported a MetS prevalence of 29% [16]. Additionally, 22% of school teachers in Ogbomoso, Nigeria, were found to have MetS [9]. Another observational research employing a cross-sectional design, conducted among 200 basic education teachers in Viçosa-MG, Brazil, found a MetS prevalence of 20% [27]. A previous cross-sectional study among secondary school teachers in Mysore, India, reported a MetS prevalence of 38.3% (115 out of 300 teachers) [28]. Using criteria from the IDF when the presence of at least 3 components of MetS was assessed among the studied teachers, more than one-fourth of the evaluated group was diagnosed with MetS.

It was noted that MetS prevalence increased with age, from 13.3% in the 40–50-year age group to 54.1% in the >50-year age group. Similarly, Monica et al. [29] reported age-specific MetS prevalence among 256 school teachers in Chennai, India. With an aging population, the increasing prevalence of MetS poses a threat due to rising rates of chronic illnesses and comorbidities [30].

In addition, the study determined that the prevalence of MetS was 26.2% in males and 25.2% in females, suggesting that the risk of developing MetS is virtually equal for both male and female teachers. Similar results were reported by Narayanappa et al. [28] in their study of MetS incidence among secondary school teachers in Mysore, India. A more recent study among the academic staff of Shahjalal University in Bangladesh found that although the difference was not statistically significant, MetS prevalence was slightly higher in female staff members (52.3%) than in males (45.8%) [31]. In the progression of MetS, gender-specific variations are not universal; rather, they are associated with social and economic status, stress related to work, body fat composition, and ethnicity, as stated in a systematic review conducted by Cornier et al. [32].

In the current study, married teachers had a statistically significant higher prevalence of MetS (26.4%) compared to unmarried teachers (20%). Supporting these findings, a study in Makassar, South Sulawesi, Indonesia, found an extremely high MetS prevalence (98%) among married school teachers [8]. In many developing nations, the likelihood of ailment may be elevated by the medical expenses related to conflictive social contacts in marriage, which can function as a substantial psychosocial stressor [33].

Undoubtedly, individuals with chronic work stress are likely to develop MetS. They face various stressful life events related to workload, finances, and health, which may be associated with insulin resistance, obesity, and other risk factors for MetS [34,35]. Consequently, the current study found a statistically significant increase in MetS prevalence (31.1%) among teachers who had worked for >20 years compared to those with ≤20 years of service (p = 0.021). The higher incidence of MetS was statistically significantly associated with the rise in the average number of days of employment/week and hours/day. Comparable conclusions were deduced from a recent investigation into the incidence of MetS among manual laborers in a manufacturing facility in the Republic of Korea [36]. Regarding the educational level of the studied teachers, those with a postgraduate degree had a statistically significant higher MetS prevalence (53.7%) compared to those with only a university degree (20.8%). This may be attributed to the considerable stress faced by teachers preparing for postgraduate studies, which likely contributed to the development of MetS.

No statistically significant variance in MetS prevalence was detected among teachers at different educational stages (primary, preparatory, or secondary). Similar results were found in a study of MetS prevalence among Indian school teachers [29]. Dealing with young students is very stressful, and teaching the complex curricula of higher stages is also challenging.

Hyperglycemia is the hallmark of T2DM, a metabolic disorder that is caused by deficiencies in either insulin secretion or activity, or perhaps both. Metabolic syndrome is a collection of cardiovascular risk factors that includes central adiposity, hypertension, elevated FBG, elevated triglycerides, and a decrease in HDL cholesterol. Insulin resistance or obesity associated with insulin resistance are the established etiologies of MetS, which is occasionally referred to as insulin resistance syndrome or syndrome X [37]. The prevalence of MetS among the diabetic teachers studied was 84.4%. Moreover, a statistically significant association was observed between FBS levels and MetS prevalence among the studied teachers. A substantial correlation between the incidence of MetS and a present and/or previous family history of T2DM among the teachers under investigation has been noted in numerous prior studies [9,27–29].

Although the mechanisms that underlie the development of MetS-related hypertension are less well understood, hypertension is a critical element of MetS. The evidence indicates that the development of numerous MetS determinants, including low-grade inflammation, insulin resistance, renin-angiotensin-aldosterone system activation, and obesity, can be influenced by elevated fructose and sodium consumption. The combination of these modifications, as well as fructose-stimulated salt absorption in the renal tubules and small intestine, can result in hypertension and a state of salt excess [38].

According to the study results, the prevalence of MetS among hypertensive teachers was 78.8%. Examination of the studied subjects revealed that those with elevated BP measurements had a statistically significant higher MetS prevalence of 51.9% compared to those with normal BP measurements. Subjects with elevated BP were either undiagnosed cases of hypertension or uncontrolled cases. Furthermore, the diagnosis of MetS was significantly linked to measured BP figures among the studied teachers. Similar previous studies emphasized the association between MetS prevalence and diagnosed cases of hypertension or even a maternal and/or paternal history of the disease among the studied teachers or academic staff [9,27–29,31].

It is noteworthy that 2 critical factors are required for the development of MetS: first, adult weight gain associated with body fat accumulation, and second, a propensity to accumulate ectopic fat in intra-abdominal organs such as the liver, pancreas, and heart. It is crucial to remember that body fat cannot be accurately predicted by BMI alone. While WC predicts body fat, it does so more accurately in terms of risk factors for cardio-metabolic diseases, as it can identify individuals with lower BMI who have higher levels of intra-abdominal fat accumulation [7]. Central obesity may decrease insulin's ability to uptake glucose, leading to insulin resistance. Tumor necrosis factor-α and interleukin-6, 2 proinflammatory adipokines produced by adipose tissue, are responsible for this process. Additionally, the activation of the renin-angiotensin system contributes to hypertension [27]. In the present study, teachers with a BMI >30 kg/m^2^ and/or increased WC had a statistically significant higher MetS prevalence compared to those with a BMI <30 kg/m^2^ and/or normal WC. In agreement with these results, Narayanappa et al. [28] reported that obesity was the most common risk factor for MetS prevalence among the studied school teachers, with a percentage of 63.3%. Another more recent study found that abdominal obesity is one of the most common components of MetS among the studied school teachers [29].

Dyslipidemia, characterized by aberrant lipid profiles, is a significant contributor to the development of MetS and is one of the primary underlying causes of CVD and T2DM in afflicted persons. The dyslipidemia that is linked to MetS is characterized by increasing levels of triglycerides, decreased levels of HDL, and increased levels of LDL. The prevalence of insulin resistance and central obesity in persons with MetS has been associated with a group of lipid profile abnormalities. Compensatory hyperinsulinemia and insulin resistance in individuals with MetS result in the increased generation of LDL particles. The relative insufficiency of lipoprotein lipase leads to decreased production of HDL particles and removal of triglyceriderich lipoproteins (TRLs) during periods of fasting and after eating. The primary lipid profile anomaly in MetS is the increased concentration of cholesteryl ester-rich fasting and postprandial TRLs. The heightened breakdown of intra-abdominal fat, which is metabolically active and releases free fatty acids into the portal circulation, is associated with resistance to insulin action or insulin deficiency. The liver metabolizes free fatty acids into triglycerides, potentially resulting in hypertriglyceridemia [39]. The study results revealed that MetS diagnosis among the studied teachers was significantly linked to the components of the lipid profile (Table 3). Similarly, dyslipidemia was significantly associated with MetS prevalence and CVD risk among school teachers in several previous studies [9,16,27–29,31].

Furthermore, smoking significantly contributes to MetS occurrence. Smoking raises the body's levels of insulinantagonistic hormones, including catecholamines, cortisol, and growth hormone. It enhances lipolysis, which increases triglyceride levels. Additionally, nicotine encourages fat breakdown, and impaired fasting glucose is believed to result from β-cell damage in the pancreas due to free fatty acids produced through these pathways [40]. In the present study, smoking was significantly associated with MetS diagnosis among the studied teachers, consistent with findings from previous studies [31,40,41]. However, no association was found in other studies [42].

The assessment of 10-year ASCVD risk among the studied teachers was estimated using the ASCVD risk score estimator per the 2018 ACC/AHA guidelines. According to the findings, the majority of the teachers in the study were at risk of developing CVD within 10 years, but with varying levels of risk: 73% had a low risk, 8.8% had a borderline risk, 15.1% had an intermediate risk, and 3.1% had a high risk (Table 2). The ASCVD risk score among the studied population was Me = 2.2 (Table 1). Several previous studies emphasized the susceptibility of school teachers to developing CVDs in the presence of MetS components and other risk factors, such as stress and unhealthy lifestyles [15,43,44].

Spearman correlation analysis in Table 4 confirmed the conclusion that MetS is a condition involving a cluster of risk factors specific to CVDs. In addition to personal and occupational risk factors, the cluster of metabolic factors includes abdominal obesity, high BP, impaired fasting glucose, and dyslipidemia. Consequently, the ASCVD risk score showed positive correlations with age, years of employment, number of working hours per day, number of working days per week, number of smoked packs of cigarettes per year, systolic blood pressure, diastolic blood pressure, BMI, WC, and serum levels of FBG, cholesterol, triglycerides, and LDL. Furthermore, a negative correlation between the ASCVD risk score and serum HDL levels was noted. Linear regression analysis revealed that the statistically significant independent predictors for ASCVD risk in this study were smoking (pack/year), duration of employment in years, and serum levels of LDL and FBG.

Finally, the prevalence of CVD and MetS among teachers in Egypt is influenced by physical inactivity, poor eating habits, and inadequate sleep as well as psychological factors like depression, anger, and anxiety which are mainly due to long working hours without leave. These factors are major risk factors that influence and predict these illnesses. Excessive workloads, low levels of professional conflict, a lack of social support at work, and a decline in professional autonomy were also found to be psychological risk factors for teachers.

## CONCLUSIONS

The prevalence of MetS among the studied school teachers was 25.8%, while the 10-year risk of developing CVDs was low for 73%, borderline for 8.8%, intermediate for 15.1%, and high for 3.1% of the teachers. Risk factors contributing to MetS included older age, marriage, longer work duration, higher educational level, increased smoking (packs per year), history of diabetes and hypertension, obesity, elevated FBG, high BP, and dyslipidemia. The study highlighted the importance of regular screening to detect these conditions early. Screening for NCDs is essential to raise awareness and motivate individuals to adopt health-promoting behaviors, such as a balanced diet and regular exercise. Additionally, school health programs should focus on helping teachers manage stress and address their health needs to create a healthy learning environment. Finally, further research with more representative samples is necessary to gain accurate insights into the prevalence of MetS and CVD risk across various subgroups of school teachers, which will assist in developing and implementing preventive strategies and interventions for high-risk populations.

### Limitations and points of strength of the study

It is essential to emphasize the limitations of this study. It employed a cross-sectional design in the first place. Nonetheless, an effort was made to calculate and randomly choose the sample, which contributed to the study's increased validity. Another drawback was the exclusion of additional variables including nutrition, exercise, and psychological aspects that may affect the MetS and CVD risk. However, one of the strengths of this work, according to the published data, was that it is the first study to determine the prevalence of MetS and associated risk of CVD among the Egyptian school teachers. The results revealed a considerable MetS prevalence and CVD risk among the studied teachers, so these findings should be verified with longitudinal, large-scale and multi-central studies.
